# INTEGRATING AND IMPROVING MULTIPLE-METHOD RESEARCH IN AGING

**DOI:** 10.1093/geroni/igae098.1489

**Published:** 2024-12-31

**Authors:** Pamela Saunders, Angela Perone

**Affiliations:** Chair; Integrating and Improving Multiple Method Aging Research; Co-Chair; Integrating and Improving Multiple Method Aging Research

## Abstract

One of the basic tenets of mixed-method and mixed-methods research methodologies is that they integrate qualitative and quantitative data to provide a more holistic understanding of a research question. This interdisciplinary symposium examines several aging research projects to illustrate best practices for integrating and improving multiple methods research projects. We draw on fields including cultural anthropology, discourse analysis, and sociocultural gerontology to examine questions of language, identity, transcription, and homelessness. In a mixed-methods study, Saunders examines the integration of data and analysis from staff interviews, residents’ conversations, and staff ratings of friendship among persons living with dementia in a long-term care setting unit. Crampton examines the value of outliers in a study of how “ old” is defined by people aging into 80+ decades. Dobbs’ paper will discuss the role of AI transcription using smartphone and web-based platform technologies to collect data from older adults and their caregivers. Perone uses a novel multi-method approach to learn about housing models that aim to prevent or intervene in homelessness among LGBTQIA+ older adults. Directors of Aging Centers Interest Group Sponsored Symposium

